# Analysis on Microstructure–Property Linkages of Filled Rubber Using Machine Learning and Molecular Dynamics Simulations

**DOI:** 10.3390/polym13162683

**Published:** 2021-08-11

**Authors:** Takashi Kojima, Takashi Washio, Satoshi Hara, Masataka Koishi, Naoya Amino

**Affiliations:** 1 Research and Advanced Development Division, The Yokohama Rubber Co., Ltd., 2-1 Oiwake, Hiratsuka 254-8601, Kanagawa, Japan; masataka.koishi@y-yokohama.com (M.K.); naoya.amino@y-yokohama.com (N.A.); 2 Department of Reasoning for Intelligence, The Institute of Scientific and Industrial Research, Osaka University, 8-1 Mihogaoka, Ibarakishi 567-0047, Osaka, Japan; washio@ar.sanken.osaka-u.ac.jp (T.W.); satohara@ar.sanken.osaka-u.ac.jp (S.H.)

**Keywords:** filled rubber, microstructure, filler morphology, molecular dynamics simulations, machine learning, convolutional neural network, persistent homology

## Abstract

A better understanding of the microstructure–property relationship can be achieved by sampling and analyzing a microstructure leading to a desired material property. During the simulation of filled rubber, this approach includes extracting common aggregates from a complex filler morphology consisting of hundreds of filler particles. However, a method for extracting a core structure that determines the rubber mechanical properties has not been established yet. In this study, we analyzed complex filler morphologies that generated extremely high stress using two machine learning techniques. First, filler morphology was quantified by persistent homology and then vectorized using persistence image as the input data. After that, a binary classification model involving logistic regression analysis was developed by training a dataset consisting of the vectorized morphology and stress-based class. The filler aggregates contributing to the desired mechanical properties were extracted based on the trained regression coefficients. Second, a convolutional neural network was employed to establish a classification model by training a dataset containing the imaged filler morphology and class. The aggregates strongly contributing to stress generation were extracted by a kernel. The aggregates extracted by both models were compared, and their shapes and distributions producing high stress levels were discussed. Finally, we confirmed the effects of the extracted aggregates on the mechanical property, namely the validity of the proposed method for extracting stress-contributing fillers, by performing coarse-grained molecular dynamics simulations.

## 1. Introduction

Filled rubber is a composite material fabricated from polymers and fine filler nanoparticles, and its mechanical properties are directly related to tire performance, such as rolling resistance, abrasion resistance, and wet traction [1,2,3,4]. To establish a relationship between the mechanical properties of the rubber and filler morphology, X-ray scattering, electronic microscopy, and atomic force microscopy studies were conducted [3,5,6,7,8,9,10,11,12,13,14]. However, these techniques could only infer their mechanical properties from the observed differences between the microstructures in the sample. Meanwhile, computer simulations can be used to visualize the internal tire region and perform virtual tire design experiments to improve its mechanical performance [15,16,17,18]. In particular, coarse-grained molecular dynamics (CGMD) simulations of polymer materials have been conducted to determine the relationship between the nanometer-scale structures observed experimentally and the meter-scale mechanical properties [19,20,21,22,23,24,25,26]. However, large-scale CGMD simulations involving hundreds of filler particles may take several weeks [25,26]. This simulation cost prevents parametric studies of filler morphology.

The best method for elucidating this relationship is based on the sampling and analysis of filler morphologies to extract the common nanostructures or features that lead to desired material properties (e.g., high elastic moduli and low hysteresis). In this regard, we are unaware of any previous experimental or theoretical studies devoted to identifying common aggregate structures among the analyzed morphologies, although some works focused on the effects produced by the filler type or volume fraction on mechanical properties [9,11,27,28,29]. Sampling a filler morphology involves creating new morphology candidates, evaluating material properties, and selecting filler morphologies that produce the desired properties. Two types of methods for generating filler morphologies have been developed: probabilistic methods, such as predicting filler positions based on the Poisson point process [13,26], and deterministic methods that directly determine each filler position [30]. However, the obtained relationship between stochastic parameters, such as the Poisson process intensity characterizing the filler morphology, and material properties is unreliable because the latter vary significantly even at the same stochastic parameters. In deterministic methods, filler morphologies are fixed by the input parameters, which represent the coordinates of each filler particle in the three-dimensional space. Nevertheless, the number of target parameters increases dramatically with the number of filler particles *n* as $O\left(3n\right)$, which prevents sampling a required number of filler morphologies. To overcome this problem, we previously developed a novel morphology-search method, which is explained in detail below [30]. To the best of our knowledge, no machine learning (ML) techniques have been applied to analyze complex filler morphologies, although ML was utilized in the material design field called “materials informatics” [31,32,33,34,35,36,37,38,39,40,41]. These studies were limited to constructing a microstructure–property linkage using ML, and did not address the mechanisms of the mechanical property. In this study, we analyzed filler morphologies leading to extremely high modulus values obtained by sampling with the ML method proposed in our previous work in order to reach a better understanding of the mechanical properties. The subsets of filler particles that induced very high stress were extracted by performing a logistic regression (LR) analysis [42] and using a convolutional neural network (CNN) [43,44]. Finally, CGMD simulations were performed to confirm the increase in stress by the extracted subsets of filler particles. As a result, it was found that anisotropic aggregate shapes and dense distributions were the keys to control the modulus values.

## 2. Problem Setting

**Figure 1:**
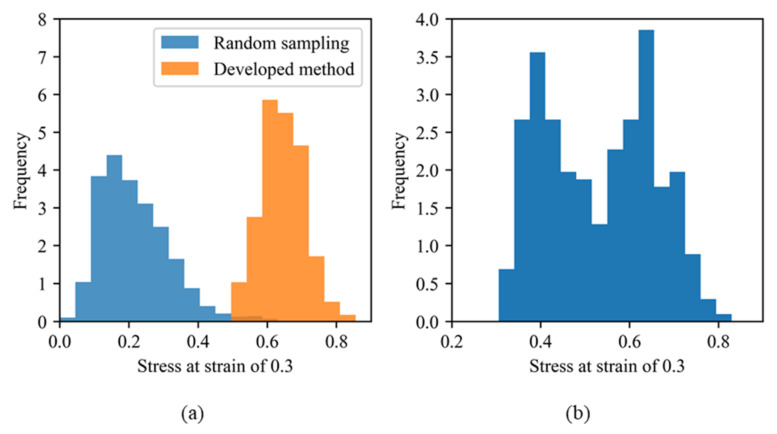
(**a**) Stress distributions obtained by the random sampling and the developed method in our preliminary study [30]. (**b**)Stress distribution of the morphologies obtained by either method with a filler volume fraction of 20 vol.% and was used as the training data in the present study.

In a preliminary study [30], we developed a search method for the filler morphology that induced a desired material property and obtained morphologies leading to extremely high stress, which could not be easily identified by random sampling (see Figure 1a; details of the utilized sampling method are provided in the next section). Stress values were calculated using the molecular dynamics simulation software packages J-OCTA/VSOP [45] and LAMMPS [46]. It is well known that filler particles percolate at filler volume fractions of 22–25 vol.% and higher, which changes the mechanical properties of the composite [47,48,49]. Moreover, the deformation of a tire during rolling reaches a strain of 0.3, and the resultant increase in stress improves the steering stability of tires [16,18]. Hence, 5000 filler morphologies (including various filler aggregates) with volume fractions of 10–20 vol.% were randomly sampled based on existing knowledge that filler aggregate enhances the stress during the first step of the project. The obtained results included only six morphologies that exceeded the stress of 0.6 at the strain of 0.3. Therefore, the objective of our previous study was to identify the morphologies providing a stress of 0.6 or higher at a filler volume fraction of 20 vol.%, which was close to the percolation threshold. The space size was 170[σ] × 170[σ] × 170[σ] (σ: unit length used in CGMD simulations), and the radius of filler particles in all morphologies was 10[σ]. As a result, 130 morphologies were obtained after three months of sampling by the developed method [30] which was a combination method of a Markov chain Monte Carlo (MCMC) [50] and a gradient descent method described in Section 3.2, and the resultant stress distribution is shown in Figure 1a.

**Figure 2:**
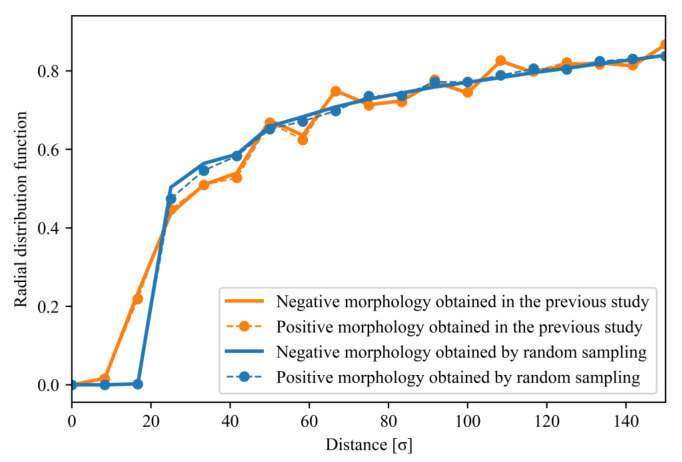
RDFs of the filler particles in the positive and negative examples. The blue lines denote the RDFs of the morphologies obtained by random sampling, and the orange lines represent the RDFs of the morphologies obtained in the previous study. The solid and dotted lines show the RDFs of the negative and positive examples, respectively.

In this study, we analyzed the features of filler morphologies obtained by random sampling and the developed sampling method [30] that provided a stress of 0.6 or higher. ML models that could classify positive and negative morphological examples were established. A positive morphological example was composed of morphologies providing a stress of 0.6 or higher at 20 vol.%, and a negative morphological example included morphologies generating a stress of 0.5 or less at 20 vol.%. The stress gap of 0.1 between the positive and negative examples was introduced to ensure clear differences between the positive and negative examples. The gap was needed to avoid subtle differences led by the continuous stress ranges of the positive/negative examples. The length of 0.1 was determined to maintain the balance of the number of positive/negative examples. Figure 1b displays the stress distribution of the filler morphologies with a filler volume fraction of 20 vol.% that was used in the present study. The radial distribution functions (RDFs) of the positive and negative examples are shown in Figure 2. These RDFs overlap over the entire region and, therefore, cannot be used to distinguish between the positive and negative examples.

## 3. Previous Work

The purpose of this work is to identify the features of filler morphologies leading to extremely high stress that can increase the steering stability of tires. To obtain reliable data, it is necessary to use a wide filler morphology space and acquire various filler morphologies. However, the high-dimensional space $O\left(3n\right)$ does not allow performing an extensive search when the coordinates of *n* particles are used as the search objective. Furthermore, morphologies that induce a desired property (for example, an extremely high modulus) are rarely found. In our previous study [30], we proposed a three-step search method for filler morphologies using a high-speed CGMD surrogate model [26]. This model briefly described in Section 3.1 was used during the search as a function that calculated stress from the filler morphology for higher search efficiency.

### 3.1. CNN-Based CGMD Surrogate Model

Training data instances were generated from the results of CGMD simulations for the randomly sampled filler morphologies. A surrogate CGMD model of filled rubber was trained using the obtained training data. Each training data instance consisted of a three-dimensional (3D) image of the filler morphology and the stress generated at a strain of 0.3. Hence, the surrogate model represented a regression model that predicted the stress at a strain of 0.3 from the filler morphology at a strain of 0.0. The surrogate model was constructed by the 3D CNN using the PyTorch [51] and included two convolutional layers and one fully connected layer. The number of kernels in both convolutional layers was equal to 50. The kernel with a size 4 × 4 × 4 moved with a stride of one in both layers. Other modeling parameters, such as the pooling size, activation function, and learning rate were described in our previous paper [26].

### 3.2. Filler Morphology Search Method

A three-step search method, which combined the CGMD surrogate model described in Section 3.1, a replica-exchange MCMC [52], a gradient descent method, and the CGMD simulation, was developed for the effective search of filler morphology in $O\left(3n\right).$ In the first step of the search process, the replica exchange MCMC was employed to discretely search among a wide range of morphologies. In the second step, the gradient descent method was applied to search for a desired morphology locally in the high-dimensional space $O\left(3n\right)$, starting from the morphologies obtained by the replica-exchange MCMC. Finally, CGMD simulations were performed to validate the obtained morphologies with the desired properties because the surrogate model used during the first two steps for higher search efficiency does not ensure the sufficiently high stress. The replica exchange MCMC employed in the first step draws samples from probability distributions by performing parallel MCMCs at different temperatures under a detailed balance. The MCMC sequence with high temperature searches a wider morphology space. In contrast, the MCMC sequence with low temperature searches locally for a rare filler morphology having the desired property. The result of the wider search conducted with the high-temperature sequence is reflected in the low-temperature sequence via the occasional exchanges between the sequences of the samples under a detailed balance. Hence, the replica exchange MCMC can mostly search for global optimal solutions. In the developed method, the filler morphology space was discretized by introducing filler candidate points x distributed in the space to increase the search efficiency, and the state of each point was controlled by the spin function s(x) defined as follows:

(1) $$s(x)=\left\{\begin{array}{cc} 1 & if\;x\;is\;filler,\\ -1 & otherwise. \end{array}\right.$$

Given that $X\subset R^{3}$ be the set of filler candidate positions, the filler morphology with u step at temperature t, $S_{u}^{t}$, is represented by $S_{u}^{t}=\left\{s\left(x\right)|x\in X\right\}\in {\left\{1,-1\right\}}^{\left|X\right|}s.t.\left|\left\{x\in X|s\left(x\right)=1\right\}\right|=N_{filler}$, where $N_{filler}$ is the number of filler particles. The filler morphology was updated during the search via the following procedure. First, a randomly selected point with $s\left(x\right)=1$ is transformed into the polymer state $s\left(x\right)=-1$. After that, a randomly selected point *y* in the polymer state, $s\left(y\right)=-1$, is changed into the filler state $s\left(y\right)=1$ if its distance from point x is d or less; thus, point y is described as follows:

(2) $$y\in X_{u}^{-}\left(x|S_{u}^{t}\right)=\{y\in X|\|y-x\|<d,s\left(y\right)=-1,s\left(y\right)\in S_{u}^{t}\}.$$

The transition probability $Q\left(S^{t}|S_{u}^{t}\right)$ from the current filler configuration $S_{u}^{t}$ to the new configuration $S^{t}=\left\{s\left(x\right)=-1,\;s\left(y\right)=1,\;s\left(z\right)\in S_{u}^{t}\;\mathrm{is}\;\mathrm{unchaged}\;\mathrm{for}\;\mathrm{all}\;z\neq x,y\right\}$ is expressed as follows:

(3) $$Q\left(S^{t}|S_{u}^{t}\right)=\frac{1}{\left|X_{u}^{-}\left(x|S_{u}^{t}\right)\right|}.$$

$S_{u+1}^{t}$ is determined by the Metropolis–Hastings algorithm under the detailed balance as follows:

(4) $$\begin{array}{c} S_{u+1}^{t}=\left\{\begin{array}{cc} S^{t} & if\;\frac{P\left(S^{t}\right)Q\left(S_{u}^{t}{|S}^{t}\right)}{P\left(S_{u}^{t}\right)Q\left(S^{t}|S_{u}^{t}\right)}>r,\\ S_{u}^{t} & otherwise \end{array}\right.| \end{array}$$

where $P\left(S^{t}\right)$ is the target distribution, and r is sampled from a uniform distribution, $P_{U}\left(\left[0,1\right]\right)$. The exchange of $S_{u}^{t}\;\mathrm{and}\;S_{u}^{t+1}$, the filler morphologies at the adjacent temperature t and t+1, was attempted in every m step under the detailed balance, where m was much larger than the relaxation periods of the corresponding sequences.

(5) randomly choose t∈T−1,

(6) $$Swap\left(S_{u}^{t},S_{u}^{t+1}\right)\;if\;\frac{P\left(S_{u}^{t+1}|t\right)P\left(S_{u}^{t}|t+1\right)}{P\left(S_{u}^{t}|t\right)P\left(S_{n}^{t+1}|t+1\right)}>r,$$

where $P\left(S_{u}^{t}|t+1\right)$ is the probability of the filler morphology $S_{u}^{t}$ at temperature t+1, and T is the total number of temperatures.

**Figure 3:**
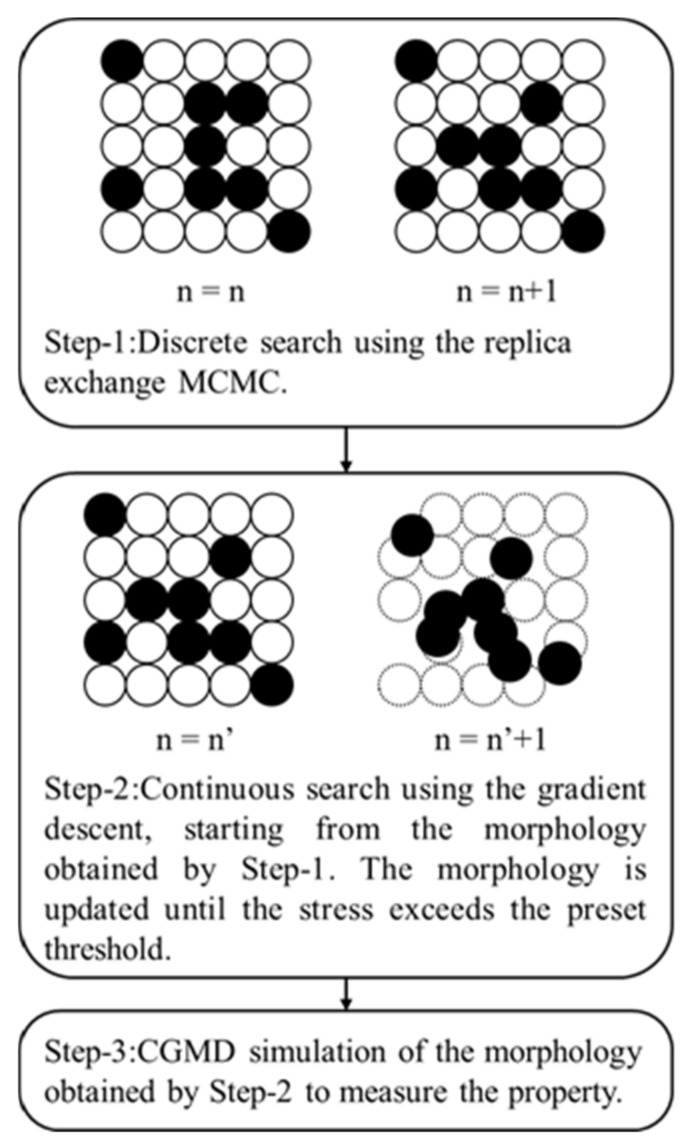
Flow chart of the developed morphology search method. The circles in Step 1 represent the filler candidate positions. The open circles and filled circles are the polymer and filler particles, respectively. The left figure in Step 2 is the initial state of this step. The right figure shows an updated filler configuration. CGMD simulation was carried out to measure the property in Step 3, because the properties evaluated in both Step 1 and Step 2 were merely predicted values by the CNN-based surrogate model for the efficient search. Thus, Step 3 was required to confirm if the morphologies obtained by the first 2 steps actually show the desired property.

In the second step, the gradient descent method was applied to search for the filler morphology locally in the high-dimensional space $O\left(3n\right)$, starting from the initial filler morphology obtained in the first step. The morphology was updated until the observed property exceeded the preset property. Finally, CGMD simulations were conducted to confirm that the obtained filler morphology actually possessed the desired properties because the properties evaluated during the search conducted using the replica-exchange MCMC and gradient descent methods were merely the values predicted by the CNN-based surrogate model. Figure 3 shows a schematic illustration of the developed filler morphology search method that combines the replica-exchange MCMC and gradient descent methods.

**Figure 4:**
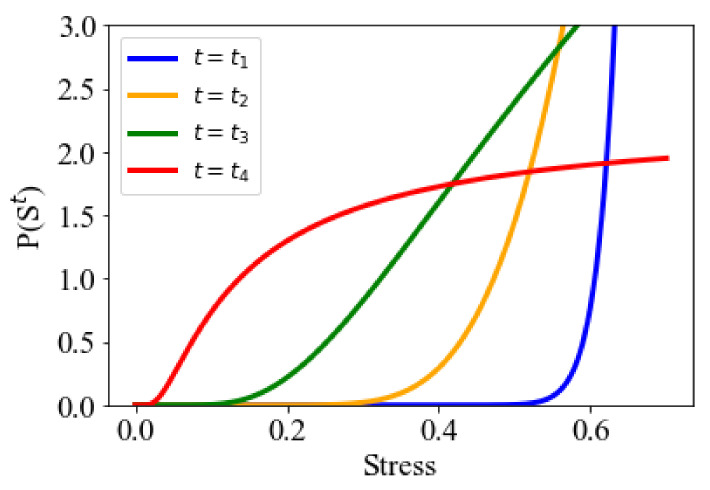
Target distributions of each temperature. The blue line, $t=t_{1}$, is the probability distribution at the lowest temperature. The red line, $t=t_{4}$, is the probability distribution at the highest temperature.

In the first sampling step of the developed method, four parallel MCMC sequences were employed. The target distribution $P\left(S^{t}\right)$ is defined as follows:

(7) $$E\left(S^{t}\right)=\frac{1}{\lambda f\left(S^{t}\right)},\;$$

(8) $$P\left(S^{t}\right)=\exp\left(-\frac{E\left(S^{t}\right)}{\alpha_{t}}\right),$$

where $f\left(\cdot \right)$ is the function that calculates the stress from the filler morphology $S^{t}$ based on the CNN-based surrogate model. The parameter $\alpha_{t}$ in Equation (8) relating temperature t to the distribution was set to $\left(\alpha_{1},\alpha_{2},\alpha_{3},\alpha_{4}\right)=\left(0.5,\;2.5,\;10,\;70\right)$. The hyperparameter λ in Equation (7), which tunes the dependency of the probability distribution on stress, was set to 1/8. Figure 4 presents the target distributions $P\left(S^{t}\right)$. Here, $P\left(S^{t_{1}}\right)$ was designed to search for filler morphologies with higher stresses, whereas $P\left(S^{t_{4}}\right)$ was designed to search a wider filler morphology space. $P\left(S^{t_{2}}\right)$ and $P\left(S^{t_{3}}\right)$ were set to largely overlap with the distributions of the other layers to introduce frequent replica exchanges between the adjacent layers for higher search efficiency. The filler morphology searches included 900,000 MCMC steps. Exchanges of filler morphologies between the adjacent temperature sequences were attempted every 3000 MCMC steps using Equation (4). Afterward, the filler morphologies obtained by the replica-exchange MCMC were updated by the gradient descent method until the stress exceeded 0.6. CGMD simulations were performed to determine the stress values of the obtained morphologies. The space size and radius of the filler particles were equal to 170[σ] × 170[σ] × 170[σ] and 10[σ], respectively, and were same as the corresponding values of the random sampling. The number of filler particles was 250, which represented the volume fraction of 20 vol.%.

## 4. Method

Two classification models were established for solving the binary classification problem: an LR-based model and a CNN-based model. In these models, a positive example includes a set of morphologies producing a stress of 0.6 or higher at a filler volume fraction of 20 vol.%. A negative example represents a set of morphologies leading a stress of 0.5 or less. Trained LR coefficients can be interpreted as the weights of all input parameters because LR is a logarithmic–linear regression model. Thus, the input parameters that influence the output can be identified based on the trained weights. A barrier to using LR for the analysis of filler morphology is the necessity of its vectorization when used as the LR input data. An example vectorization is to arrange the pixel values of an imaged filler morphology in one dimension. However, a drawback of this vectorization method is its vulnerability to image conversion. For example, the vectorized image of the original image differs from the vectorized image of a rotated image because each vector component does not correspond to the pixels of the same fillers. This difference implies that an image conversion operation (such as rotation) changes the parameters or features of the input data. Thus, LR using a straightforward vectorization cannot evaluate the effect of each input parameter on the output stress. To avoid this problem, this study employs a devised approach that filler morphologies were quantified by a persistent homology (PH) and vectorized by constructing a persistence image (PI), then the obtained vectors were used as the input parameters [53,54,55,56]. In contrast, morphology quantification is not required when a CNN-based classification model is employed because it can extract systemic features of the input images through their convolutions. In this study, a CNN-based classification model, which outputs a probability that an imaged filler morphology of input data is positive, was established to extract the filler particles producing a strong influence on mechanical properties. This CNN-based model that consisted of a convolutional layer of the imaged filler morphology could extract the filler particles related to the objective mechanical properties in the image.

### 4.1. LR and PH Analyses

**Figure 5:**
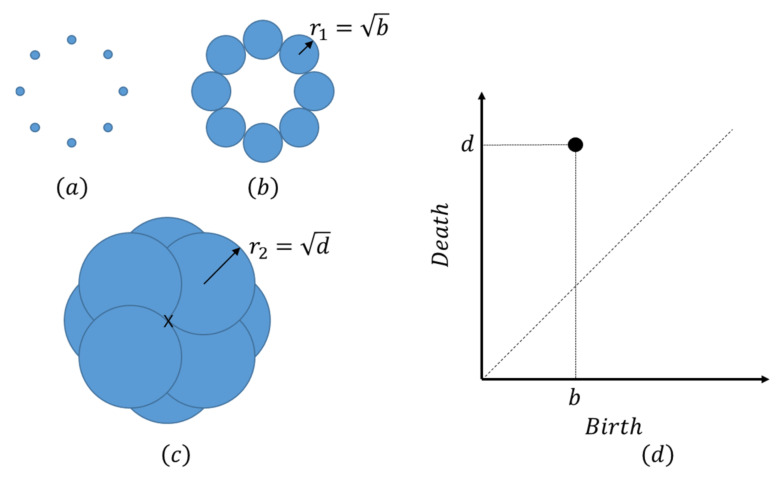
Schematic diagram illustrating the PH. (**a**) The balls are disconnected when the ball radius r is smaller than $r_{1}$. (**b**) A loop emerges at $r=r_{1}=\sqrt{b}$. (**c**) The loop disappears at $r=r_{2}=\sqrt{d}$. (**d**) PD representing the results of PH analysis.

Filler morphologies were quantified by PH. PH is a mathematical method for investigating the hidden structure in complex data and has been applied previously to analyze polymerized materials [55]. Let us assume that there are k balls at $z_{1},z_{2},\cdots ,z_{k}$ as shown in Figure 5a. In this study, each filler particle is represented by a ball with radius r. k=250 is the total number of filler particles, and $z_{i}$ denotes the coordinate of the center of the i-th filler particle. All balls are disconnected at small values of r, as shown in Figure 5a. As r increases to $r_{1}$, the balls coalesce to give “birth” to a loop, as shown in Figure 5b. Furthermore, the empty space enclosed by the loop is completely covered by balls at $r=r_{2}$; in this case, the balls meet at the “death” position denoted by the cross mark in Figure 5c, and the loop disappears. This example shows that a loop has a “birth” length $b=r_{1}^{2}$, and a “death” length $d=r_{2}^{2}$, and that it exists only when $b<r^{2}<d$. PH is the map corresponding to the region spanning from $\left\{z_{1},z_{2},\cdots ,z_{k}\right\}$ to $\left\{\left(b_{1},d_{1}\right),\left(b_{2},d_{2}\right),\cdots ,\left(b_{l},d_{l}\right)\right\}$, where l is the number of loops formed by k filler particles in a space. The result of the PH analysis is visualized in the scatter plot of $\left(b_{i},d_{i}\right)$ depicted in Figure 5d, which represents a persistent diagram (PD).

The PD on $R^{2}$ is converted into a vector using the PI. First, $PD=\left\{\left(b_{i},d_{i}\right)|i=1,\cdots ,l\right\}$ is mapped onto the persistence surface ρ as follows:

(9) $$\rho \left(x,y\right)=\overset{l}{\underset{i=1}{\sum }}w\left(b_{i},d_{i}\right)\exp\left(-\frac{{\left(b_{i}-x\right)}^{2}+{\left(d_{i}-y\right)}^{2}}{2h^{2}}\right)$$

(10) $$w\left(b,d\right)=\arctan\left(\beta {\left(d-b\right)}^{\gamma }\right).$$

**Figure 6:**
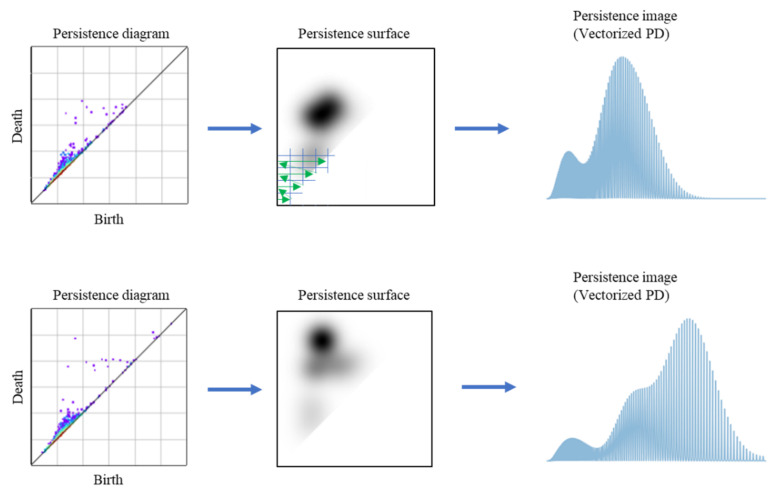
Schematic illustration of the vectorization procedure. The left plots include the PDs; the center figures are the persistence surfaces; and the right histograms contain the PIs. Two morphologies are vectorized in the upper and lower figure panels. As shown in the upper center figure, the persistence surface is divided by a grid, and representative values of all grid cells are arranged along the green arrows.

Here, β>0, h>0, and γ>0 are the parameters. $w\left(b,d\right)$ and h are the weight function and standard deviation of the Gaussian distribution, respectively. Any function can be used as a weight function. In this study, to emphasize the characteristic loops with long lifetimes (the difference between the death length and the birth length), the arctan function used in previous studies was employed as the weight function [57]. γ was set to 3. The persistence surface was discretized into g×g subdomains. The discretized persistence surface was reduced to a finite-dimensional vector by arranging the integrated values over the subdomain. The subdomains of the upper left half of the surface with distributed $\left(b_{i},d_{i}\right)$ were arranged in such a way that their birth and death occurred in the ascending order. The vectorized PD is the PI. Figure 6 describes the vectorization procedure and resultant PIs of the two morphologies, which significantly differ from each other.

The obtained dataset consisted of the PIs of the positive/negative examples and stress-based classes determined by the CGMD. The trained classification model predicting the probability p of the positive example was constructed using LR as follows:

(11) $$logit\left(p\right)=\ln\left(\frac{p}{1-p}\right)=\overset{l}{\underset{i=0}{\sum }}\omega_{i}PI_{i}$$

where ω is the weight of each *PI* component and is a parameter to be trained. *l* is the number of *PI* components in the upper left part of the discretized persistence surface, which is defined as follows:

(12) $$l=\overset{g}{\underset{i=1}{\sum }}i$$

**Figure 7:**
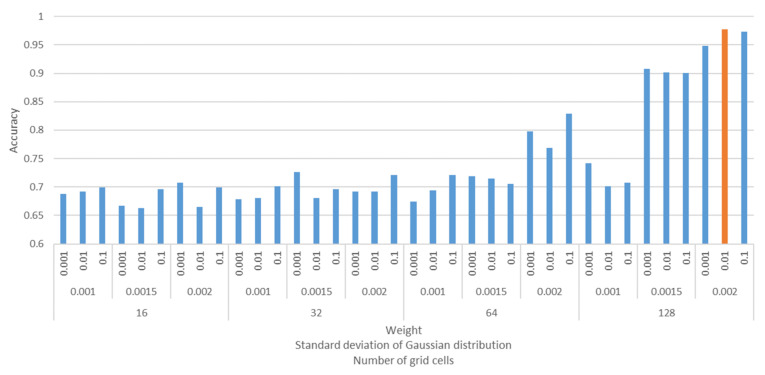
Prediction accuracies of the validation data with different combinations of parameters. The accuracy was defined as the ratio of the number of morphologies that predicted the correct class to the number of validation data instances. The top row in the horizontal axis is the weight on the persistence surface β; the middle row is the standard deviation of the Gaussian distribution h; and the bottom row is the number of subdomains g. The orange bar represents the combination of parameters that produces the highest accuracy.

An L1 regularization term with c=1.0 was added to the loss function to avoid overfitting. L1 regularization can reduce the dimension of ω by applying zeros to multiple regression coefficients. PD regions that dominate the output were identified based on the trained nonzero ω values, and the filler particles related to the positive/negative example were predicted by extracting the loops corresponding to the PD regions. In this study, PH analysis was performed under periodic boundary conditions similar to those of the CGMD. The obtained dataset was divided into training data, validation data, and test data with a ratio of 8:1:1. The optimal parameters were determined by estimating the accuracy of the classification model trained by the training dataset with respect to the validation data not used for training. The prediction accuracy was measured by applying test data not used for training and validation. The parameters to be trained were h in Equation (9), β in Equation (10), and g in Equation (12). The best combination of these parameter values was found from $h=\left\{0.001,0.0015,0.002\right\}$, $\beta =\left\{0.001,0.01,0.1\right\}$, and $g=\left\{16,32,64,128\right\}$ by a grid search algorithm. Figure 7 shows the prediction accuracies estimated by the validation data. The combination $\left(h,\beta ,g\right)=\left(0.002,0.01,128\right)$ provided the highest accuracy, and this combination gave the same level of accuracy for the test data.

**Figure 8:**
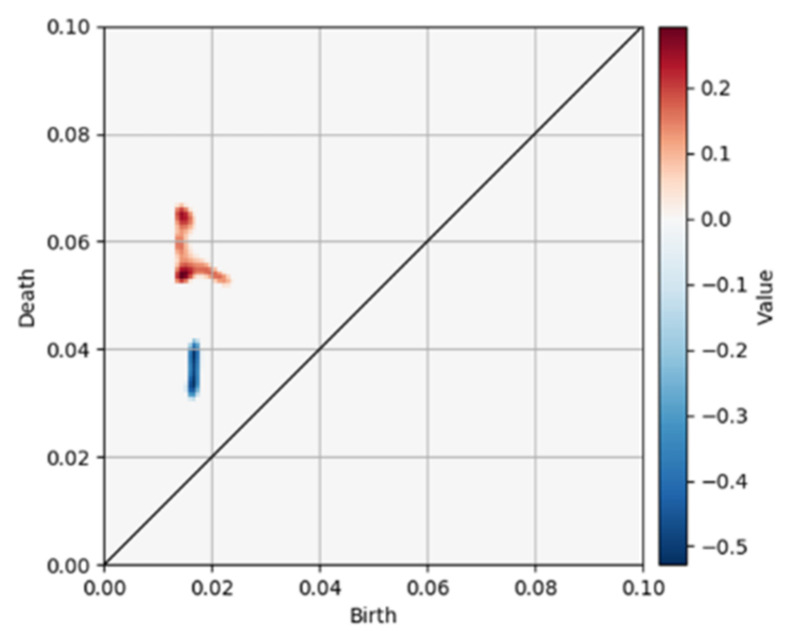
Distribution of the L1 regularization LR coefficient, which predicts a class from the filler morphology. The coefficient is visualized in the PD space. The red region corresponds to the positive coefficients and contributes to the positive example. The blue region corresponds to the negative coefficients and contributes to the negative example.

**Figure 9:**
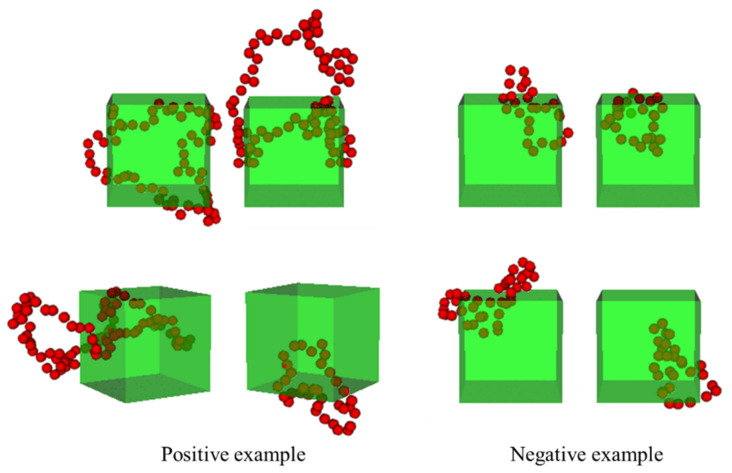
Examples of the loops composed of filler particles, which were extracted by the PH. The green box denotes the modeled space, and the red dots represent the filler particles.

Figure 8 shows the distribution of the trained LR coefficient in the PD space. According to Figure 6, PI, an LR input vector, was generated by converting the PD representing the finite dataset $\left\{\left(b_{i},d_{i}\right)|i=1,\cdots ,l\right\}$ into the continuous data (persistence surface) and discretized. Thus, the trained coefficient, ω, can be interpreted as the weight for each region of the PD. The PD region corresponding to $PI_{i}$ where the regression coefficient is positive, contributes to the positive example. In contrast, the PD region corresponding to $PI_{i}$ where the regression coefficient is negative contributes to the negative example. The region between (birth, death) = (0.015, 0.05–0.07) and (birth, death) = (0.018, 0.03–0.04) contribute to the positive and negative examples, respectively (Figure 8), and some loops extracted from both regions are presented in Figure 9. The loops larger than the modeled space size result from the PH performed under the periodic boundary conditions. We will compare the loops extracted from each class in the next section.

### 4.2. CNN-Based Analysis

**Figure 10:**
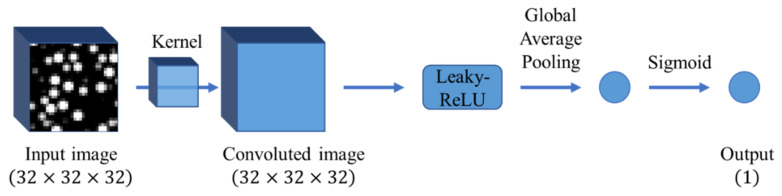
CNN architecture utilized in this work. The numbers in parentheses represent the dataset sizes. The input image of 32×32×32 pixels is convoluted into the image of 32×32×32 pixels under the periodic boundary conditions with a single kernel. The leaky ReLU function and global average pooling were applied to the convoluted image to reduce the dimension. Finally, the probability of the positive example is output using the sigmoid function.

The CNN input data consist of a filler morphology image; therefore, morphology quantification is not required. The regions contributing to the positive and negative examples are extracted by analyzing the sensitivity of each pixel of the input image to the output using the trained classification model. Thus, the fillers in the extracted regions were predicted to be strongly related to the mechanical properties of filled rubber. However, the trained classification model is a “black box” and is hardly interpretable. Hence, extracting the dominant region (as was done by LR) employing a CNN with a complex architecture is difficult. To alleviate this issue, the simplest CNN, which contained only a convolutional layer without a fully connected layer, was utilized in this work (Figure 10). Here, the input image was translated into the output by the CNN via the following procedure. First, the input image was convoluted by only one filter. Second, a leaky rectified linear unit (leaky ReLU) [58] and global average pooling [59] were applied to the convoluted image for the reduction into a scaler. Finally, the predicted class was output through a sigmoid function. Therefore, the predicted output class was a positive example when the average of the pixel values of the convoluted image was positive. The negative example was predicted when the average of the pixel values was negative; therefore, judgment rules could be determined easily. In addition, the regions of the input image that dominate the output class can also be estimated from the coordinates of the pixels with positive/negative values in the convoluted image. Note that batch normalization was performed to stabilize the training after the leaky ReLU [60,61]. The kernel moved with a stride of one in the convolutional layer under the periodic boundary conditions so that both the center and the edge of the input image could be evaluated at the same weight, and the size of the convoluted image was equal to the size of the input image. Therefore, the filler particles in the input image corresponding to the convoluted image area with positive pixel values can be easily extracted as the fillers contributing to the positive example by simply superimposing the convoluted image onto the input image.

**Figure 11:**
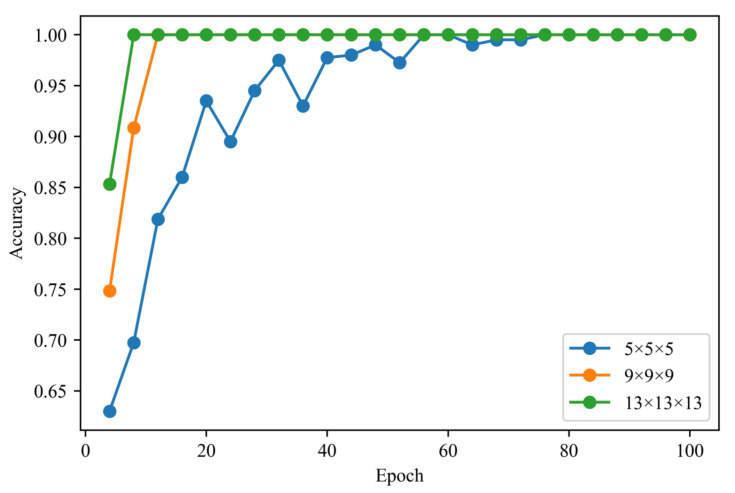
Relationships between the epoch number and prediction accuracy determined by applying the validation data. The prediction accuracy was equal to the ratio of the number of morphologies that predicted the correct class to the number of validation data instances. The blue line, orange line, and green line correspond to the prediction accuracies of the kernels with sizes of 5×5×5, 9×9×9, and 13×13×13, respectively.

To investigate the relationship between the kernel size and prediction accuracy, the prediction accuracies achieved at kernel sizes of 5×5×5, 9×9×9, and 13×13×13 were compared. The dataset consisted of an input image with a size of 32×32×32 (Figure 10) and a stress-based class. The dataset was divided into training data, validation data, and test data with a ratio of 8:1:1 similarly to the dataset used for LR in Section 4.1. The batch size and learning rate were 50 and 0.0005, respectively. Adam was used as the optimization algorithm [62]. The prediction accuracy was measured every four epochs by applying validation data not used for training. The resultant CNN learning curves are shown in Figure 11. The prediction accuracies of all kernel sizes at an epoch of 100 were equal to 1.0. The prediction accuracies measured using the test data were also 1.0.

**Figure 12:**
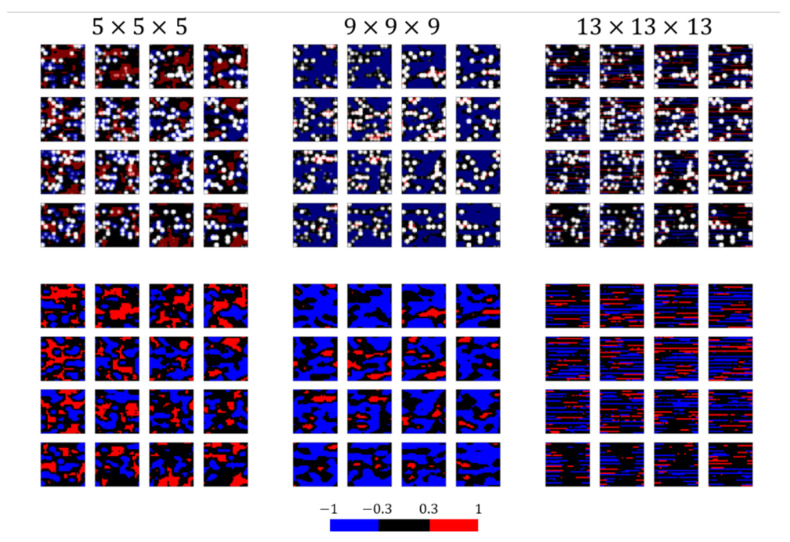
Input and convoluted images obtained using the kernels with sizes of 5×5×5, 9×9×9, and 13×13×13. The upper row represents the superimposed images of input and convoluted images. The white circles and black areas denote the filler particles and rubber regions in the input images, respectively. The red and blue areas are visualized in the convoluted images depicted in the lower row and represent the pixels with positive and negative values, respectively. The red areas contribute to the positive example, and the blue areas contribute to the negative example. The pixels with values ranging from −0.3 to 0.3 are visualized in black to emphasize the areas with large contributions.

Figure 12 shows a comparison between the input image and the convoluted image, which is passed through the filter. The sizes of both images were 32×32×32, and 16 cross-sectional images of every 2 pixels in depth were presented. Because the elastic modulus of the filled rubber is roughly proportional to the filler volume fraction, the region contributing to the positive example (the pixels of the convoluted image with positive values) preferably matches a partial region of the filler in the input image. The pixels with positive values of the convoluted image obtained using the kernel with a size of 9×9×9 matched the filler of the input image. In contrast, the red regions where the positive pixel values of the convoluted image with a kernel size of 5×5×5 were located in the rubber region of the input image, and a striped pattern was constructed in the convoluted image using the kernel with a size of 13×13×13. The number of pixels and the space size of the input image were 32×32×32 and 170[σ] × 170[σ] × 170[σ], respectively; thus, approximately 5.3[σ] was imaged with a pixel. Hence, four pixels were required to image the filler particle with the radius $r=10\left[\sigma \right]$ used in this study. Therefore, the kernel with a size of 5×5×5 could not convolute all information about filler aggregates (including connections with the adjacent filler particles) because the kernel convoluted only one filler particle. Accordingly, the CNN predicted the class by evaluating the rubber region rather than the filler aggregates, and the pixels with positive values in the convoluted image were distributed across the rubber region. Meanwhile, the kernel with a size of 9×9×9 convoluted the space including two fillers along the X-, Y-, and Z-directions. This implies that this kernel evaluated the structure of filler aggregates by convoluting the information of the adjacent filler particles. This procedure resulted in a good match between the pixels with positive values in the convoluted image and the filler particles in the input image. The striped pattern was generated because the change in the convoluted area by the large kernel with a size of 13×13×13 and stride of one was very small.

**Figure 13:**
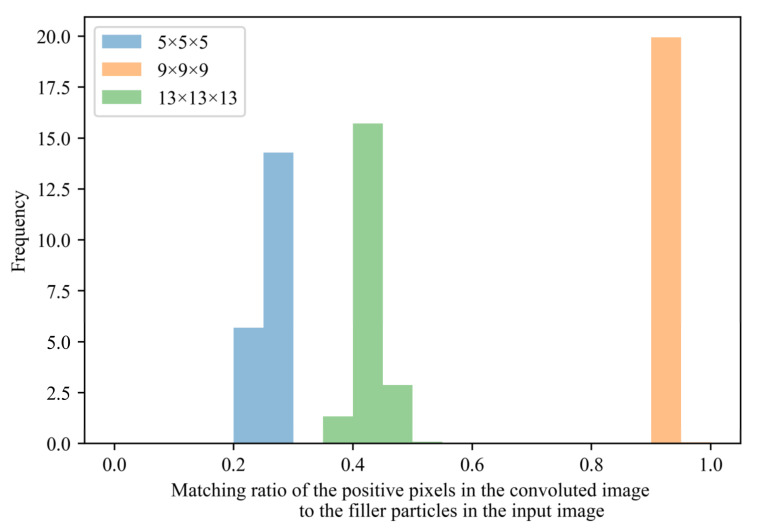
Ratio of the positive pixels in the convoluted image to the filler particles in the input image, which was defined as an A/B fraction. Here, A is the number of pixels with positive values in the convoluted image that overlap with the filler particles in the input image. B is the total number of pixels with positive values in the convoluted image. The blue, orange, and green colors correspond to the kernel sizes 5×5×5, 9×9×9, and 13×13×13, respectively.

Figure 13 shows the histograms of the fraction of positive pixels in the convoluted image geometrically matching the filler particles in the input image. All morphologies of the positive samples were used for its construction. The positive pixels of the image convoluted by the kernel with a size of 9×9×9 matched the filler particles in the input image by more than 90%. This result illustrates that the kernel size of 9×9×9 is appropriate to clarify the filler aggregate–property linkage.

## 5. Results and Discussion

### 5.1. Filler Aggregates Extracted by the PH and CNN Methods

**Figure 14:**
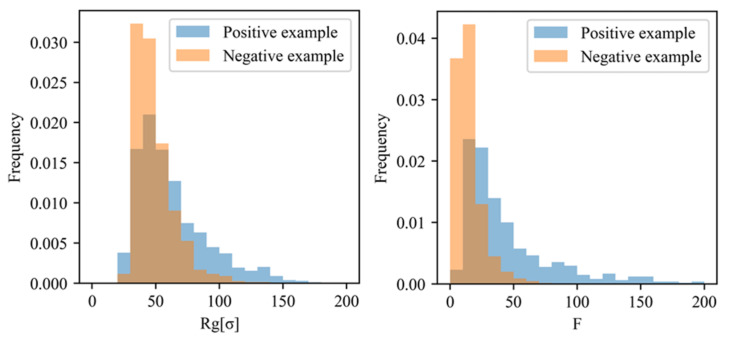
Histograms of the radius of gyration, $R_{g}$, and the number of filler particles in the loop, F, of the filler aggregate extracted by the PH. The blue bars and orange bars denote the aggregates extracted from the positive and negative examples, respectively.

First, the loops consisting of the filler particles extracted by PH from all morphologies in the positive and negative examples were compared based on the regression coefficients of LR with L1 regularization presented in Section 4.1. As described above, the positive example included the morphologies providing a stress of 0.6 or higher, and the negative example contained the morphologies providing a stress of 0.5 or less at a filler volume fraction of 20 vol.%. The radius of gyration defined in Equation (13), $R_{g}$, and the number of filler particles, F, were determined for both examples. $r_{g}$ and $r_{n}$ in Equation (13) are the center of gravity of the loop and the coordinate of the filler particle contained in this loop, respectively. In this study, the loop extracted by the PH was considered a filler aggregate because the loop represented a set of filler particles from the PH perspective. Figure 14 indicates that both $R_{g}$ and F values of the positive example are apparently larger than those of the negative example, suggesting that the positive example included larger aggregates than those of the negative example.

(13) $$R_{g}=\sqrt{\frac{1}{F}\overset{F}{\underset{n=1}{\sum }}{\left(r_{n}-r_{g}\right)}^{2}}$$

(14) $$r_{g}=\frac{1}{F}\overset{F}{\underset{n=1}{\sum }}r_{n}$$

**Figure 15:**
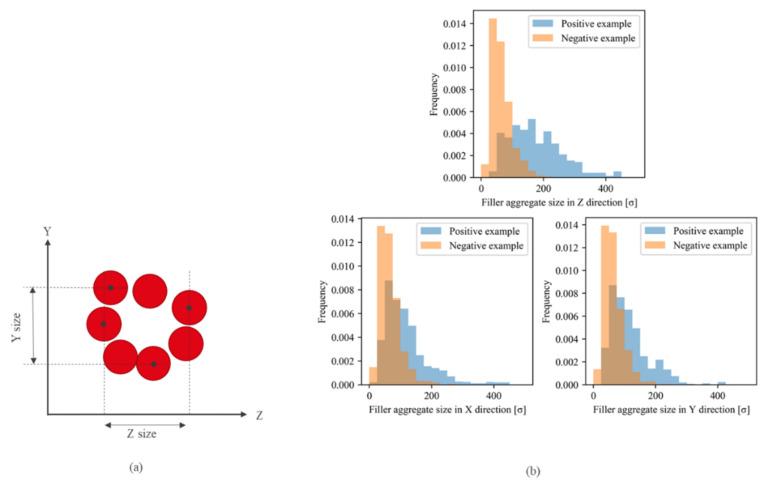
(**a**) Definition of the aggregate size. The red circles and black points represent the filler particles and their centers, respectively. The size in each direction is the difference between the largest and smallest coordinates along this direction. (**b**) Aggregate size distribution in each direction. The blue and orange bars denote the results obtained for the positive and the negative examples, respectively. Z-direction is the elongation direction of the system, while X- and Y-directions are compressed during deformation.

The aggregate sizes were compared in all directions to investigate the shape difference between the aggregates extracted from the positive and negative examples (Figure 15). The aggregates extracted from the positive example were larger than those extracted from the negative example in all directions. Specifically, the difference in aggregate size in the Z-direction (the elongation direction of the system) between the positive and negative examples was apparently larger than those in the X- and Y-directions. Hence, the morphological characteristics leading to a higher stress in the positive example were larger size in all directions, especially along the deformation direction.

**Figure 16:**
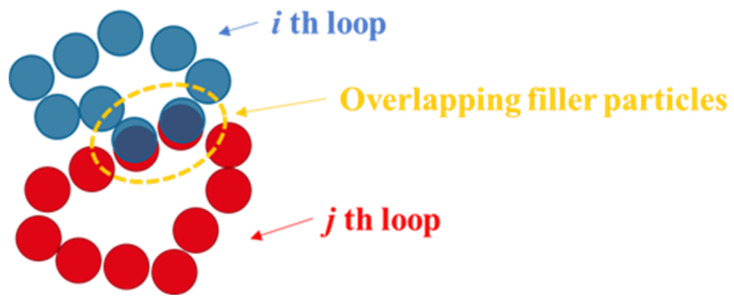
Schematic illustration of the filler particles belonging to multiple loops. The blue and red circles denote the filler particles constituting different loops. The particles surrounded by the orange circle belong to both the *i*-th and *j*-th loops connected physically. However, these two loops are considered different aggregates.

Note that PH did not impose a restriction on the overlapping constituent particles of the loop. Therefore, some filler particles were extracted as constituents of different loops (Figure 16). In this study, the loop extracted by the PH was considered a filler aggregate, as described above. This means that the i-th and j-th loops in Figure 16 represent different aggregates, although they were connected physically with the filler particles surrounded by the orange circles. On the other hand, it was reported in some studies that the stress induced in the rubber region between the filler aggregates that were considerably deformed during the deformation process was the main source of stress in the entire system [12,25,63]. This conclusion implies that the distribution of filler aggregates may be a key factor affecting the mechanical properties of filled rubber. However, it is difficult to measure the spatial distribution of aggregates using PH loops because these aggregates easily overlap with each other.

**Figure 17:**
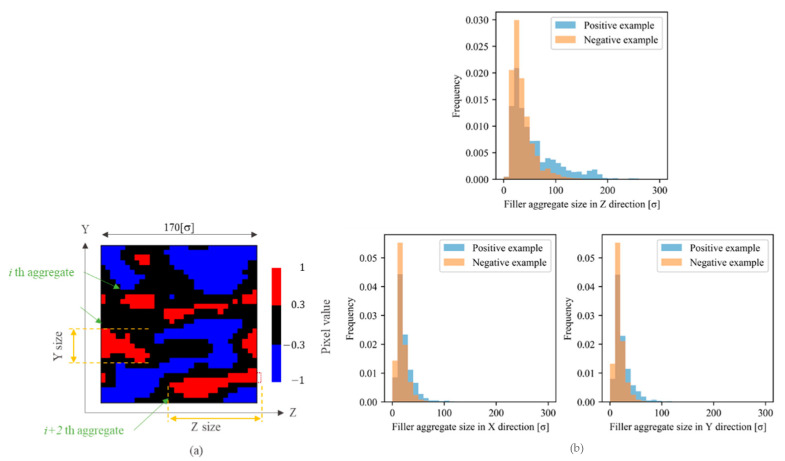
Example of the aggregate extracted by the CNN and size distributions of filler aggregates. (**a**) Cross-section of the convoluted image. The aggregate size is defined as the difference between the largest and smallest pixel coordinates. The red region is the set of pixels with positive values that contributes to the positive example. The blue region is the set of pixels with negative values that contributes to the negative example. The black region includes pixels with small values ranging from −0.3 to 0.3 and does not affect the output. The adjacent red pixels are extracted as filler aggregates under the periodic boundary conditions. (**b**) Histograms of the size of the set of pixels with positive values, namely aggregate sizes, obtained from all morphologies in the positive (blue bars) and negative (orange bars) examples. Z is the elongation direction of the system.

Next, the filler morphologies were analyzed using the CNN. In this study, the simplest CNN consisting of a convolutional layer with a kernel was employed, as described in Section 4.2. This CNN was able to easily extract the filler particles contributing to the stress based on the pixel values of the convoluted image. The pixels with positive values of the convoluted image passing through the filter with the size 9×9×9 match the filler particles in the input image (Figure 13). Therefore, a set of the adjacent pixels with positive values in the convoluted image was considered a filler aggregate (Figure 17a). Pixels with a value of 0.3 or higher were extracted as they strongly contributed to the positive example. In this study, filler aggregates were represented by the filler particles located in the area contributing to the positive example of the convoluted image, although the aggregate in a broad sense also included the filler particles located in the neighborhood of the extracted area. The aggregate size was measured in each direction as the difference between its largest and smallest coordinate (Figure 17a) similar to the PH analysis. Figure 17b displays the distributions of filler aggregate sizes, which indicate that the aggregates extracted from the positive example were larger than those extracted from the negative example. In particular, the size difference in the Z-direction (the elongation direction of the system), was greatly similar to the PH data. However, the results presented in Figure 15b indicate that the sizes of the aggregates extracted by the PH were larger than those of the aggregates extracted by the CNN in all directions. This difference resulted from the nature of these algorithms utilized for extracting filler aggregates by these two techniques: the PH could extract larger aggregates than CNN because some filler particles constituted multiple PH loops.

**Figure 18:**
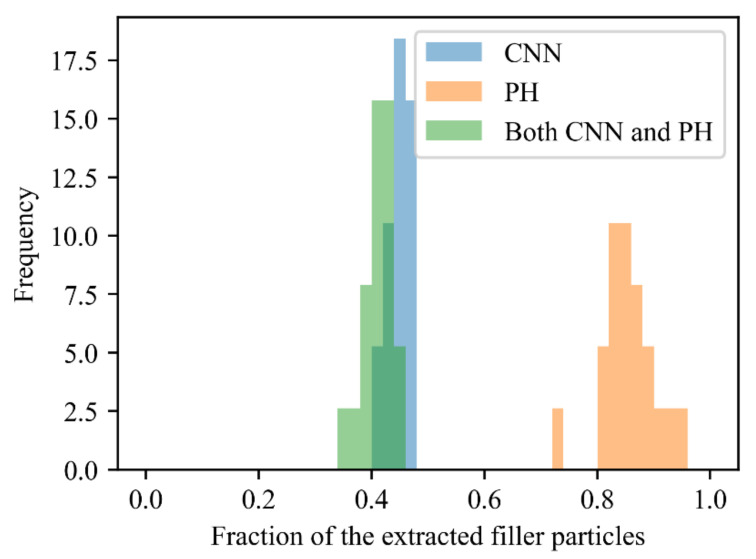
Fractions of the filler particles extracted by the PH and CNN that contributed to the positive example. Their values were computed as the ratios of the numbers of pixels of the extracted particles to the number of pixels for all filler particles. The blue bars denote the ratios of the filler particles extracted by CNN; the orange bars represent the PH results; and the green bars denote ratios of the filler particles common to the fillers extracted by CNN and the fillers extracted by PH.

The aggregates extracted from all morphologies in the positive example by the PH and those extracted by the CNN were compared, because the aggregate extracted by both methods could be considered a core structure that strongly increases the stress and constitutes the positive example. The results presented in Figure 18 indicate that 80% or more of filler particles were extracted by the PH. However, only 40% of such particles were extracted by the CNN. The histogram of the filler particles extracted by the CNN almost overlaps the histogram of the particles extracted by both the CNN and PH. Hence, the filler particles extracted by the CNN were considered a subset of the particles extracted by the PH and presumed to strongly contribute to the mechanical properties of filled rubber.

**Figure 19:**
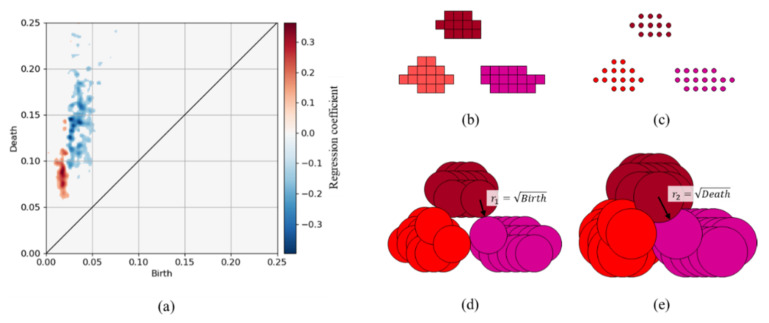
Distribution of the LR coefficient of the aggregate extracted by the CNN on the PD, and schematic illustration of the filler aggregates extracted by the PH method. (**a**) Distribution of the L2 regularized LR coefficient, which reflects the distribution of aggregates. The regression coefficient is positive in the red region and negative in the blue region, which contribute to the positive and negative examples, respectively. (**b**) Aggregates extracted by the CNN. Each rectangle represents a pixel, and each color denotes a filler aggregate. (**c**) Centers of the pixels that constitute filler aggregates. (**d**) $r_{1}$ is a representative value of the distance between the filler surfaces because a loop emerges at $r=r_{1}=\sqrt{Birth}$. (**e**) $r_{2}$ is a representative size of the filler agglomerate (quadratic aggregate) because the loop disappears at $r=r_{2}=\sqrt{Death}$.

In addition, the difference between the distributions of the filler aggregates in the positive and negative examples extracted by the CNN was investigated because the CNN-extracted aggregate was a subset of the PH-extracted aggregate and could be considered a core structure, as described above, and some studies reported that the filler aggregate distribution was a factor determining the mechanical property [2,3,12]. The positions of the filler aggregates extracted by the CNN represented by the coordinates of the pixels with positive values were analyzed by the PH. The resultant PDs were vectorized by the PI, as described in Section 4.1. The LR-based classification model was trained using the dataset that consisted of the PI and class labels. The positive example included the morphologies producing a stress 0.6 or higher, and the negative example contained the morphologies generating a stress of 0.5 or less, same as thus far. The parameters to be trained were h in Equation (9), β in Equation (10), and the number of subdomains g in Equation (12). The best combination of these parameters $\left(h,\beta ,g\right)=\left(0.002,0.1,256\right)$ was searched on a grid of $h=\left\{0.001,0.0015,0.002\right\}$, $\beta =\left\{0.001,0.01,0.1\right\}$, and $g=\left\{16,32,64,128,256\right\}$. The prediction accuracies measured by applying the validation data and test data were equal to approximately 0.8. Note that L2 regularization with the regularization parameter c=1.0 was employed during training because the purpose of this investigation was not a dimension reduction. Figure 19a shows the distribution of the trained LR coefficients on the PD, while Figure 19b–e display the schematic diagrams of the PH for filler aggregates distribution. The “birth” label on the horizontal axis represents the distance between the adjacent filler aggregates or the surface distance between aggregates (see Figure 19d). The “death” label on the vertical axis represents the size of the aggregate set called an agglomerate or quadratic aggregate (Figure 19e). Figure 19a illustrates that both the birth and death of the positive example were smaller and distributed in a narrower region than those of the negative example. This indicates that the distances between the aggregate surfaces and the agglomerate sizes of the positive example were small. However, both the birth and death of the negative example were large and distributed in a relatively wide region, which explained that the aggregates were dispersed in the rubber region for the negative example.

Based on the all results described in this Section 5.1, the main characteristics of the morphologies that induce an extremely high stress are summarized as follows.

- Anisotropic aggregate shapes (clusters of spherical filler particles). The larger aggregates (especially in the elongation direction) induce higher stress values. These characteristics are illustrated in Figure 15 and Figure 17.
- The dense distribution of filler aggregates reflected the short distances between their surfaces. In addition, the sizes of agglomerates (quadratic aggregates) are small as shown in Figure 19.

### 5.2. Comparison between the Extracted and Non-Extracted Filler Particles

**Figure 20:**
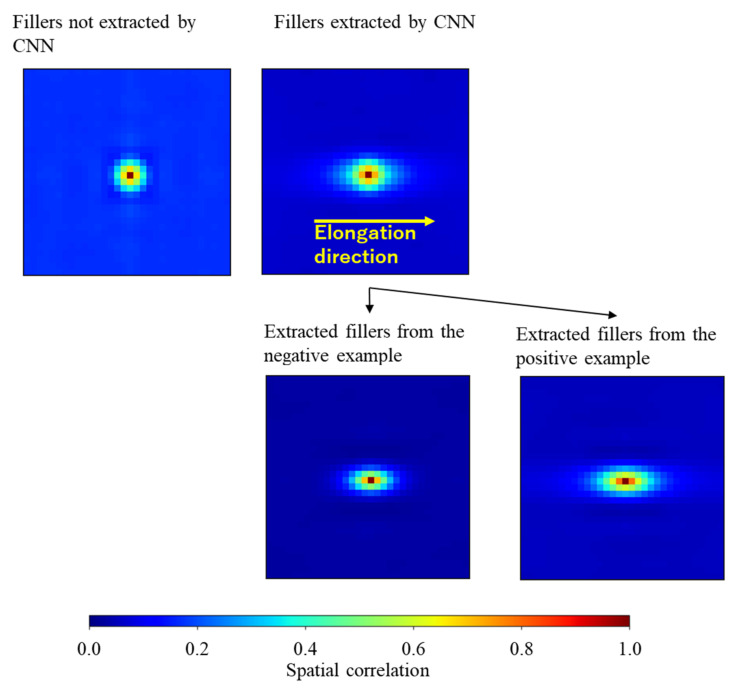
Spatial correlations in the YZ plane of r-space including $r=\left(0,0,0\right)$ of the CNN-extracted fillers from all morphology examples that contribute to the high stress and that of the non-extracted fillers (upper row figures). The isotropic spatial correlation of the fillers not extracted by the CNN suggests the isotropically shaped filler aggregates. Meanwhile, the anisotropic spatial correlation of the filler particles extracted by the CNN indicates that the probability of filler orientation along the elongation direction is high. Furthermore, bottom two figures show the spatial correlations of the extracted fillers from all positive examples and all negative examples, respectively. The higher orientation probability of the filler particles extracted from the positive example along the elongation direction suggests that the aggregate size measured along the elongation direction in the positive example is larger than the size of the negative example.

The extracted filler particles contributing to the positive example and the not-extracted fillers were compared under the aim to lead to a filler morphology producing a higher stress than the positive example by increasing the filler fraction extracted by the CNN. The filler morphologies were compared using the two-point spatial correlation $L\left(r\right)$ defined by Equation (15) [34,64,65,66], where r was a direction vector. $L\left(r\right)$ was equivalent to the probability that the material of a starting point of r was same as the material of an end point of r.

(15) $$L\left(r\right)=\frac{1}{\rho \left|V\right|}\underset{x\subset V}{\sum }m_{x}m_{x+r}$$

(16) $$m_{x}=\left\{\begin{array}{c} 1\;if\;the\;material\;of\;x\;is\;filler,\\ 0\;otherwise\; \end{array}\right.$$

where V, x, and $m_{x}$ are the set of the location vectors of all pixels in the images, a location vector of a pixel, and material of a pixel located at x, respectively. $\left|V\right|$ is the volume of V. ρ is either the volume fraction of the filler particles extracted by the CNN in V or the volume fraction of filler particles not extracted in V. It was not used in the standard $L\left(r\right)=\frac{1}{\left|V\right|}\underset{x\subset V}{\sum }m_{x}m_{x+r}$ employed in some studies [34,64,65,66,67]. In this study, $L\left(r\right)$ of the extracted and the non-extracted filler particles must be normalized using their volume $\rho \left|V\right|$, respectively. The upper left and upper right figures in Figure 20 show $L\left(r\right)$ averaged over the fillers non-extracted and extracted from all morphology examples, respectively. The bottom left and bottom right figures depict $L\left(r\right)$ averaged over the fillers extracted from all morphologies in the negative and positive examples, respectively. All $L\left(r\right)$ were computed under the periodic boundary condition in the range of $-85\left[\sigma \right]\leq r_{i}\leq 85\left[\sigma \right]$, where $r_{i},\left(i=X,Y,Z\right)$ was the orthogonal coordinate of r. Note that the cell size was 170[σ] × 170[σ] × 170[σ], thus the probability of finding the same material in the half size of the cell was computed as $L\left(r\right)$. Both $L\left(r\right)$ of filler particles extracted from the positive examples and $L\left(r\right)$ of filler particles extracted from the negative examples (presented in the bottom row of Figure 20) exhibit an anisotropic distribution with high probability along the elongation direction. This anisotropic distribution indicated that the aggregate size along the elongation direction was larger than the aggregate sizes in the other directions, which was consistent with the aggregate characteristics described previously. Meanwhile, $L\left(r\right)$ of the filler particles not extracted by the CNN (see the upper left figure of Figure 20) exhibit an isotropic distribution representing the isotropic aggregate shapes such as spheres. Therefore, it was concluded that the reinforcement effect of the isotropically shaped aggregates was not strong, and that the anisotropically shaped aggregates with larger sizes along the elongation direction strongly influenced the produced stress.

### 5.3. Validation Using CGMD Simulations

**Figure 21:**
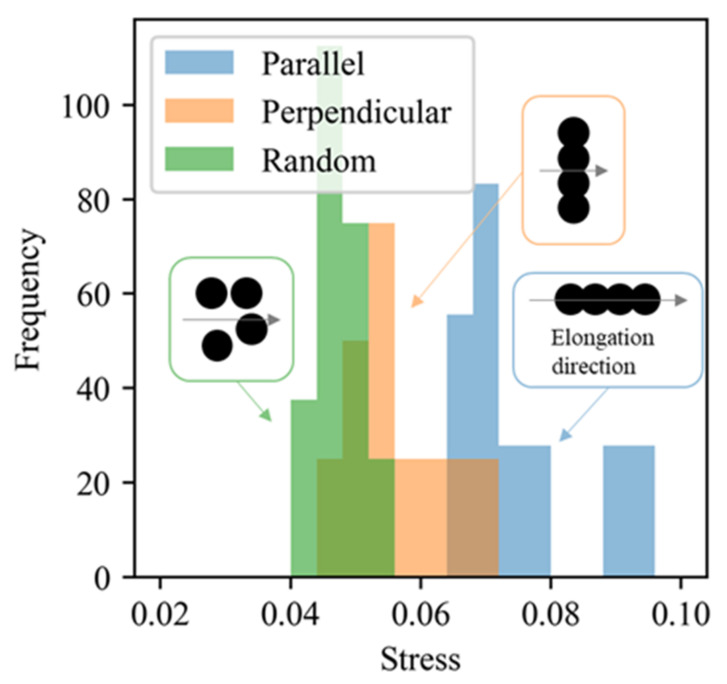
Distributions of the CGMD-simulated stress at a strain of 0.3. The blue bars denote the results obtained for the CNN-extracted aggregates from the positive example. The Z-direction was the elongation direction. The orange bars represent the elongation of the CNN-extracted filler aggregates along the X- and Y-directions. The elongation direction was perpendicular to the aggregate principal direction (Z) in these cases. The green bars denote the results obtained for the filler configurations, in which the particle positions were randomly determined with the filler volume equal to that for the blue bars. Schematic diagrams of the filler aggregates and elongation direction are shown in the rectangles with the frame colors identical to those of the corresponding bars. The black circles and arrows designate the filler particles and the elongation direction, respectively.

CGMD simulations were performed to assess if the CNN extracted filler particles actually contributed strongly to the mechanical properties and provided the high stress. For this purpose, the difference of stresses at the strain of 0.3 generated by the CNN extracted fillers were compared with the stress generated by the same number of randomly arranged filler. Ten morphologies were randomly selected from the positive example of which morphologies provided the stress of 0.6 or higher. The space size of the morphologies, the radius of filler particles, and the filler volume fraction were 170[σ] × 170[σ] × 170[σ], 10[σ], and 20 vol.%, respectively, as defined in Section 2. All filler particles except the ones extracted by the CNN were replaced with polymers in each morphology to create a new filler morphology consisting of the fillers generating the high stress only. Note that these extracted filler aggregates were oriented along Z-direction in all of the filler morphologies. Extra ten filler morphologies consisting of the randomly arranged fillers with the polymers were also generated as our previous study [25]. CGMD simulations on these twenty filler morphologies under stretching to a strain of 0.3 along the Z-direction were performed. In addition, CGMD simulations of the CNN extracted filler morphologies stretching along the X- and Y-directions were also performed to investigate the relationship between the elongation directions and filler aggregate shapes. The simulation conditions, such as the interaction between the filler and polymer and elongation rate, were identical to those utilized in our previous study[25]. Figure 21 shows the stress distributions that resulted from these CGMD simulations. A comparison between the blue and green bars in Figure 21 revealed that the stresses generated by the CNN-extracted aggregates were larger than the stresses produced by the randomly arranged filler particles during the elongation along the Z-direction. This result confirmed the validity of the proposed method for extracting filler aggregates that would likely contribute to the mechanical properties and for clarifying the relationship between the anisotropic shape of these aggregates and the mechanical properties. A comparison of the blue and orange bars in Figure 21 shows that the produced stress increases when the elongation direction matches the principal direction of the filler aggregates. This result suggests that the connection of filler particles along the elongation direction has a stronger impact on the reinforcement effect than the collision of filler particles induced during the deformation along the X- and Y-directions.

## 6. Conclusions

We analyzed the features of filler morphology producing an extremely high modulus by ML to better understand the mechanical properties of filled rubber and identify new approaches to their effective control. For this purpose, we formulated a binary classification problem for the CGMD measured stress at a strain of 0.3 and investigated the resultant filler morphologies by two different methods. First, filler morphology was quantified by the PH and PI, and an LR-based classification model was developed. The filler aggregates that strongly contributed to the stress were extracted based on the coefficients of the trained classification model. Second, a CNN was applied to establish another classification model. The aggregates contributing to stress generation were extracted based on the image convoluted by the kernel. It was found that the aggregate size along the elongation direction was an important factor affecting stress generation because both methods extracted the filler aggregates whose sizes in the elongation direction were larger than those in the other directions. It was also found that the larger aggregates induced higher stress values. In addition, the CNN-based classification model revealed that the dense aggregate distribution (the short distance between aggregates) was another important factor influencing the stress value. These results, that the anisotropy shape of the aggregate and deformation along the primary direction of the aggregate were the keys to increase the stress, were confirmed by the CGMD simulations.

In the present study, the filler morphologies with a volume fraction of 20 vol.% were examined. In future works, we will investigate the morphologies with a larger volume fraction leading to filler percolation. We will design the new filler morphologies reflecting the keys. Moreover, the properties not considered in this study (such as hysteresis) and morphologies composed of multiple filler types will be analyzed as well. The method used in this work can be widely applied to other microstructured materials and may strongly contribute to the advancement of materials science in various fields.

## Data Availability

Not applicable.
